# Supplementary material for: OpenMM 7: Rapid development of high performance algorithms for molecular dynamics
Source: PLoS Comput Biol. 2017 Jul 26;13(7):e1005659. doi: 10.1371/journal.pcbi.1005659 (PMC5549999; doi:10.1371/journal.pcbi.1005659)
Supplement: S1 Source Code — (ZIP) [file pcbi.1005659.s002.zip › OpenMM-7.0.1-Source/docs/api-python/generated/simtk.openmm.openmm.AmoebaOutOfPlaneBendForce.html]

AmoebaOutOfPlaneBendForce — OpenMM 7.0.1.dev-5e86c4f documentation


# AmoebaOutOfPlaneBendForce¶

*class* `simtk.openmm.openmm.``AmoebaOutOfPlaneBendForce`(*\*args*)¶
:   This class implements the Amoeba out-of-plane bend interaction.

    To use it, create an OutOfPlaneBendForce object then call addOutOfPlaneBend() once for each outOfPlaneBend. After an out-of-plane bend has been added, you can modify its force field parameters by calling setOutOfPlaneBendParameters(). This will have no effect on Contexts that already exist unless you call updateParametersInContext().

    `__init__`(*self*) → AmoebaOutOfPlaneBendForce¶
    :   \_\_init\_\_(self, other) -> AmoebaOutOfPlaneBendForce

        Create an AmoebaOutOfPlaneBendForce.

    Methods

    |  |  |
    | --- | --- |
    | `__init__`((self) -> AmoebaOutOfPlaneBendForce) | \_\_init\_\_(self, other) -> AmoebaOutOfPlaneBendForce |
    | `addOutOfPlaneBend`((self, particle1, ...) | Add an out-of-plane bend term to the force field. |
    | `getAmoebaGlobalOutOfPlaneBendCubic`(...) | Get the global cubic term |
    | `getAmoebaGlobalOutOfPlaneBendPentic`(...) | Get the global pentic term |
    | `getAmoebaGlobalOutOfPlaneBendQuartic`(...) | Get the global quartic term |
    | `getAmoebaGlobalOutOfPlaneBendSextic`(...) | Get the global sextic term |
    | `getForceGroup`((self) -> int) | Get the force group this Force belongs to. |
    | `getNumOutOfPlaneBends`((self) -> int) | Get the number of out-of-plane bend terms in the potential function |
    | `getOutOfPlaneBendParameters`(self, index) | Get the force field parameters for an out-of-plane bend term. |
    | `setAmoebaGlobalOutOfPlaneBendCubic`(self, cubicK) | Set the global cubic term |
    | `setAmoebaGlobalOutOfPlaneBendPentic`(self, ...) | Set the global pentic term |
    | `setAmoebaGlobalOutOfPlaneBendQuartic`(self, ...) | Set the global cubic term |
    | `setAmoebaGlobalOutOfPlaneBendSextic`(self, ...) | Set the global sextic term |
    | `setForceGroup`(self, group) | Set the force group this Force belongs to. |
    | `setOutOfPlaneBendParameters`(self, index, ...) | Set the force field parameters for an out-of-plane bend term. |
    | `updateParametersInContext`(self, context) | Update the per-bend term parameters in a Context to match those stored in this Force object. |
    | `usesPeriodicBoundaryConditions`((self) -> bool) | Returns whether or not this force makes use of periodic boundary conditions. |

    `getNumOutOfPlaneBends`(*self*) → int¶
    :   Get the number of out-of-plane bend terms in the potential function

    `setAmoebaGlobalOutOfPlaneBendCubic`(*self*, *cubicK*)¶
    :   Set the global cubic term

        |  |  |
        | --- | --- |
        | Parameters: | **cubicK** (*double*) – the cubic force constant for the angle |

    `getAmoebaGlobalOutOfPlaneBendCubic`(*self*) → double¶
    :   Get the global cubic term

        |  |  |
        | --- | --- |
        | Returns: | global cubicK term |
        | Return type: | double |

    `setAmoebaGlobalOutOfPlaneBendQuartic`(*self*, *quarticK*)¶
    :   Set the global cubic term

        |  |  |
        | --- | --- |
        | Parameters: | **quarticK** (*double*) – the quartic force constant for the angle |

    `getAmoebaGlobalOutOfPlaneBendQuartic`(*self*) → double¶
    :   Get the global quartic term

        |  |  |
        | --- | --- |
        | Returns: | global quartic term |
        | Return type: | double |

    `setAmoebaGlobalOutOfPlaneBendPentic`(*self*, *penticK*)¶
    :   Set the global pentic term

        |  |  |
        | --- | --- |
        | Parameters: | **penticK** (*double*) – the pentic force constant for the angle |

    `getAmoebaGlobalOutOfPlaneBendPentic`(*self*) → double¶
    :   Get the global pentic term

        |  |  |
        | --- | --- |
        | Returns: | global penticK term |
        | Return type: | double |

    `setAmoebaGlobalOutOfPlaneBendSextic`(*self*, *sexticK*)¶
    :   Set the global sextic term

        |  |  |
        | --- | --- |
        | Parameters: | **sexticK** (*double*) – the sextic force constant for the angle |

    `getAmoebaGlobalOutOfPlaneBendSextic`(*self*) → double¶
    :   Get the global sextic term

        |  |  |
        | --- | --- |
        | Returns: | global sexticK term |
        | Return type: | double |

    `addOutOfPlaneBend`(*self*, *particle1*, *particle2*, *particle3*, *particle4*, *k*) → int¶
    :   Add an out-of-plane bend term to the force field.

        |  |  |
        | --- | --- |
        | Parameters: | - **particle1** (*int*) – the index of the first particle connected by the outOfPlaneBend - **particle2** (*int*) – the index of the second particle connected by the outOfPlaneBend - **particle3** (*int*) – the index of the third particle connected by the outOfPlaneBend - **particle4** (*int*) – the index of the fourth particle connected by the outOfPlaneBend - **k** (*double*) – the force constant for the out-of-plane bend |
        | Returns: | the index of the out-of-plane bend that was added |
        | Return type: | int |

    `getOutOfPlaneBendParameters`(*self*, *index*)¶
    :   Get the force field parameters for an out-of-plane bend term.

        |  |  |
        | --- | --- |
        | Parameters: | **index** (*int*) – the index of the outOfPlaneBend for which to get parameters |
        | Returns: | - **particle1** (*int*) –   the index of the first particle connected by the outOfPlaneBend - **particle2** (*int*) –   the index of the second particle connected by the outOfPlaneBend - **particle3** (*int*) –   the index of the third particle connected by the outOfPlaneBend - **particle4** (*int*) –   the index of the fourth particle connected by the outOfPlaneBend - **k** (*double*) –   the force constant for the out-of-plane bend |

    `setOutOfPlaneBendParameters`(*self*, *index*, *particle1*, *particle2*, *particle3*, *particle4*, *k*)¶
    :   Set the force field parameters for an out-of-plane bend term.

        |  |  |
        | --- | --- |
        | Parameters: | - **index** (*int*) – the index of the outOfPlaneBend for which to set parameters - **particle1** (*int*) – the index of the first particle connected by the outOfPlaneBend - **particle2** (*int*) – the index of the second particle connected by the outOfPlaneBend - **particle3** (*int*) – the index of the third particle connected by the outOfPlaneBend - **particle4** (*int*) – the index of the fourth particle connected by the outOfPlaneBend - **k** (*double*) – the force constant for the out-of-plane bend |

    `updateParametersInContext`(*self*, *context*)¶
    :   Update the per-bend term parameters in a Context to match those stored in this Force object. This method provides an efficient method to update certain parameters in an existing Context without needing to reinitialize it. Simply call setOutOfPlaneBendParameters() to modify this object’s parameters, then call updateParametersInContext() to copy them over to the Context.

        The only information this method updates is the values of per-bend term parameters. The set of particles involved in a term cannot be changed, nor can new terms be added.

    `usesPeriodicBoundaryConditions`(*self*) → bool¶
    :   Returns whether or not this force makes use of periodic boundary conditions.

        |  |  |
        | --- | --- |
        | Returns: | true if nonbondedMethod uses PBC and false otherwise |
        | Return type: | bool |

    `__delattr__`¶
    :   x.\_\_delattr\_\_(‘name’) <==> del x.name

    `__format__`()¶
    :   default object formatter

    `__getattribute__`¶
    :   x.\_\_getattribute\_\_(‘name’) <==> x.name

    `__hash__`¶

    `__reduce__`()¶
    :   helper for pickle

    `__reduce_ex__`()¶
    :   helper for pickle

    `__sizeof__`() → int¶
    :   size of object in memory, in bytes

    `__str__`¶

    `getForceGroup`(*self*) → int¶
    :   Get the force group this Force belongs to.

    `setForceGroup`(*self*, *group*)¶
    :   Set the force group this Force belongs to.

        |  |  |
        | --- | --- |
        | Parameters: | **group** (*int*) – the group index. Legal values are between 0 and 31 (inclusive). |

High performance molecular simulation on GPUs


### Navigation

- Application Layer
- Library Layer
  - Core Objects
  - Forces
  - Integrators
  - Extras

©2015, Stanford University and the Authors.
|
Powered by Sphinx 1.3.1
& Alabaster 0.7.7
|
Page source
